# Susceptibility to hypoxia and breathing control changes after short-term cold exposures

**DOI:** 10.3402/ijch.v72i0.21574

**Published:** 2013-08-05

**Authors:** Lyudmila T. Kovtun, Mikhail I. Voevoda

**Affiliations:** 1 Federal State Budgetary Institution “Research Institute of Physiology and Fundamental Medicine” under the Siberian Branch of the Russian Academy of Medical Sciences, Novosibirsk, Russia; 2 Research Institute of Internal and Preventive Medicine of the Siberian Branch of RAMS, Novosibirsk, Russia

**Keywords:** susceptibility to hypoxia, hypercapnic ventilatory response, hypoxic ventilatory response, cold exposure, thermoregulation

## Abstract

**Background:**

Hypoxia is the reduction of oxygen availability due to external or internal causes. There is large individual variability of response to hypoxia.

**Objective:**

The aim of this study was to define individual and typological features in susceptibility to hypoxia, its interrelation with hypoxic and hypercapnic ventilatory responses (HVR and HCVR, respectively) and their changes after cold acclimation.

**Design:**

Twenty-four healthy men were tested. HVR and HCVR were measured by the rebreathing method during hypoxic and hypercapnic tests, respectively. These tests were carried out in thermoneutral conditions before and after cold exposures (nude, at 13°C, 2 h daily, for 10 days). Susceptibility to hypoxia (sSaO_2_) was determined as haemoglobin saturation slope during hypoxic test.

**Results:**

It was found that HVR and HCVR significantly increased and susceptibility to hypoxia (sSaO_2_) tended to decrease after cold acclimation. According to sSaO_2_ results before cold exposures, the group was divided into 3: Group 1 – with high susceptibility to hypoxia, Group 2 – medium and Group 3 – low susceptibility. Analysis of variances (MANOVA) shows the key role of susceptibility to hypoxia and cold exposures and their interrelation. Posterior analysis (Fisher LSD) showed significant difference in susceptibility to hypoxia between the groups prior to cold acclimation_,_ while HVR and HCVR did not differ between the groups. After cold acclimation, susceptibility to hypoxia was not significantly different between the groups, while HCVR significantly increased in Groups 1 and 3, HVR significantly increased in Group 3 and HCVR, HVR did not change in Group 2.

**Conclusions:**

Short-term cold exposures caused an increase in functional reserves and improved oxygen supply of tissues in Group 1. Cold exposure hypoxia has caused energy loss in Group 3. Group 2 showed the most appropriate energy conservation reaction mode to cold exposures. No relation was found between the thermoregulation and the susceptibility to hypoxia.

Hypoxia is usually defined as the reduction in oxygen availability due to external or internal causes. External hypoxic factors include climbing to high altitude, air travel and breathing gas mixtures with low oxygen content. Internal factors include exercise, cardiovascular and respiratory diseases, and increased metabolic demand such as the maintenance of body temperature in cold conditions. Susceptibility to hypoxia shows the functioning level of the gas transportation system and measures body functional potential.

The process of gas exchange involves all systems and organs of the body down to the cellular level. Our model is structured as 3 interrelated compartments: respiratory, cardiovascular systems and blood, which provide a level of state variables PaO_2_, PaCO_2_, pH, excess or deficiency of buffer bases (Base Excess, BE). Each compartment is also self-regulated. This set of interactions is reflected in the level of haemoglobin saturation (SaO_2_). It is the reason why SaO_2_ is the convenient parameter for estimating the hypoxic susceptibility (1–3).

Large interindividual variations of responses to hypoxia have to be expected (1), especially in individuals with pre-existing diseases (4). Some authors suggest that such an evident variability is a biological need for maintaining the heterogeneity of a population (5). The therapeutic or training effects of intermittent hypoxia depend on the individual response. Therefore, there is a demand to refine training methods in order to improve tolerance to hypoxia, considering the individual hypoxia susceptibility level.

In the mountains, at higher elevations, in addition to hypoxia there is also exposure to changes in temperature. In low-temperature conditions, the respiratory system is influenced by conflicting factors. On the one hand, ventilation should be increased to provide oxygen demand for heat production. On the other hand, hypoventilation is needed to reduce heat losses associated with breathing (6). In these conditions, the respiration control should provide optimal performance of breathing. Consequently, it can be assumed that changes in a ventilation control initiate respiratory system cold acclimation.

Human respiratory control can be regarded as an open, adaptive and multilevel automatic system where PaCO_2_, PaO_2_ and pH variables deviation are controlled. Hypercapnic stimulus is regarded as the main one. Hypercapnic/hypoxic ratio in the breathing control is estimated at 7:1 (7). In response to CO_2_ increasing in the inspired air, ventilation increases linearly. In response to lower inspired oxygen, ventilation varies on the hyperbolic curve and remains almost unchanged until the partial pressure of O_2_ is reduced to 50–85 mm Hg (8, 9). This depends on individual susceptibility to hypoxia. Hypercapnic ventilatory response (HCVR) determines the individual pulmonary ventilation level (10, 11). HCVR and hypoxic ventilatory response (HVR) are genetically determined state variables (12, 13). Nevertheless, they can be changed under the influence of the factors that affect the partial pressure of blood gases (PaO_2_ and PaCO_2_), such as the hypoxic and/or the hypercapnic gas mixes inhaled, breathing exercises, diseases causing additional breathing resistance and cold exposure.

There is evidence that some people have reduced HVR. These are high-altitude ethnic groups, divers and patients with chronic obstructive pulmonary disease. It is known that short-term exposure to high-altitude hypoxia increases HVR (14) and HCVR (15) in healthy humans, and after acclimation to hypoxia they decrease.

Chemosensitivity during cold exposure is a complicated phenomenon. The difficulty lies in distinguishing changes in the actual chemoreceptors sensitivity and the ventilatory response reduction associated with the cold receptors inhibitory effect directly on the respiratory centres.

It is important to find out what changes in respiratory control occur under cold and hypoxia.

The aim of our study was to define individual and typological features in susceptibility to hypoxia, its interrelation with hypoxic and hypercapnic ventilatory responses and their changes after cold exposures.

## Materials and methods

Twenty-four healthy male volunteers aged 17–24 years and inhabitants of West Siberia participated. Informed consent was obtained after the experimental protocol was explained to all subjects. The Bioethics Committee of the Institute of Physiology approved the study. Study design is shown on Table I.

**Table I:** Design of experiment with cold exposure

Days	Conditions of measurement	Measured parameters
0	Thermoneutral (26°C)	Anthropometric data, gas exchange, hypoxic ventilatory response, hypercapnic ventilatory response
1	Cold exposure (2 h, 13°C)	The core temperature (Tre), the temperature of the skin (Tsk) and the body (Tb), contractile activity of skeletal muscle, the heat debt
1–9	Cold exposure (2 h, 13°C)	
10	Cold exposure (2 h, 13°C)	The core temperature (Tre), the temperature of the skin (Tsk) and the body (Tb), contractile activity of skeletal muscle, the heat debt
11	Thermoneutral (26°C)	Anthropometric data, gas exchange, hypoxic ventilatory response, hypercapnic ventilatory response

Initially ventilatory chemosensitivity to CO_2_ and O_2_ was defined. HCVR was measured following a rest period during 30 min in thermoneutral conditions (26°C) according to rebreathing Read technique (16). Minute ventilation VE (L/min) and the end-tidal CO_2_–FetCO_2_ (vol.%) were determined. HCVR was analyzed by plotting VE vs. FetCO_2_ and expressed as slope S from regression equation: VE=B+S*FetCO_2_. After the subjects relaxed for 1 h, the HVR was determined by the rebreathing method by Weil et al. (17). VE, O_2_, CO_2_ and oxyhaemoglobin saturation (SaO_2_) were registered. HVR was analyzed by plotting VE vs. PAO_2_ to a hyperbolic curve: VE=V_0_+A/(PAO_2_-32), where PAO_2_ is alveolar O_2_ tension in mmHg, A is proportional to the ventilatory response to hypoxia vigour. Susceptibility to hypoxia was determined as saturation slope (sSaO_2_) during hypoxic test: SaO_2_=SaO_2(0)_-sSaO_2_*time. Eos Sprint “Erich Yaeger” gas analyzer (Germany) and pulsoximeter “Criticare” (USA) were used.

Subjects were exposed to the cold in a climatic chamber for 2 h each day at 13°C for 10 days. They were dressed in swimming trunks and lying down on steel for 2 h. Before the cold test, the subjects rested for 60 min in thermoneutral conditions (26°C). The rectal temperature (Tre) and the mean skin temperature (Tsk) were measured for the next 30 min. During the following next 2 h while subjects were exposed to cold, Tre and Tsk were measured. All testing procedure was repeated in the tenth cold exposure.

Tsk was determined as skin temperature of forehead, chest, thigh, hand and calf according to the formula: Tsk=0.5Tchest+0.2Tthigh+0.18Tcalf+0.07Tforehead+0.05Thand (°C). Heat debt (HD) was determined.

Body temperature (Tb) and HD were determined:

$$\begin{array}{c} \text{Tb}=\text{0.79Tre}+\text{0 .21Tsk (}{}^{\circ}\text{C )}-\text{for thermoneutral conditions.}\\ \text{Tb}=0.67\text{Tre}+0.33\text{Tsk (}{}^{\circ}C)-\text{for cold test.}\\ \text{HD}=\text{(Tre}*\text{mass}*\text{s .47(kJ}), \end{array}$$

 where Tre is the total change in Tre (in °C) (18)

Data were analyzed using Student's *t*-test for paired samples and the analysis of variance (MANOVA), regression analysis for S of HCVR and SaO_2_. Data were expressed as the mean±SE.

## Result

As a result of 10 days of 2-h daily cold exposures, rectal temperature (Tre) decreased significantly during the cold test on the tenth day [37.47±0.048(°C) in the thermoneutral conditions vs. 37.03±0.07(°C) at 120 min of cold test (P<0.0001)]; skin temperature (Tsk) was the same at 120 min of cold test on day 10 as on the 1st day: 27.99±0.17(°C) vs. 27.99±0.21(°C). HD tended to decrease. As considered by Bittel (19), all of these are the main acclimation criteria. Decreasing of T_re_ during cold test after cold acclimation, the same T_sk_ at the end of cold tests in the first and tenth days are signs of hypothermic isoinsulative general cold acclimation.

Isoinsulative hypothermic cold acclimation development was accompanied by a significant increase in hypercapnic and hypoxic ventilatory responses in thermoneutral conditions: HCVR – 1.66±0.09 before cold exposures vs. 1.99±0.14 after cold acclimation (P=0.04); HVR – 114.5±11.6 before cold exposures vs. 155.0±14.1(P=0.03) after cold acclimation. Increasing chemoreceptor sensitivity to O_2_ and CO_2_ serves to improve O_2_ supply under low-temperature conditions. Similar changes of breathing control occur under hypoxia (14, 15).

Individual basic susceptibility to hypoxia was determined as saturation slope (sSaO_2_) during hypoxic test before cold exposures. It is necessary to emphasize that the interrelation between the thermoregulation parameters and susceptibility to hypoxia was not found.

The subjects were divided into 3 groups to find individual typological differences on both susceptibility to hypoxia and ventilatory response to O_2_ and CO_2_ before and after cold exposures. According to sSaO_2_ results before cold exposures, the group was divided into 3 by percentile. Those whose results were in the lower 25th percentile, that is, with high susceptibility to hypoxia composed Group 1, n=6; those in the 25–75th percentile, that is, with medium susceptibility – Group 2, n=12; and those over 75th percentile, that is, with low susceptibility – Group 3, n=6. Values of sSaO_2_: −4.09±0.3; −2.34±0.07 and−1.4±0.18, respectively. Before cold exposure, the selected groups did not differ on the HVR and HCVR.

**Fig. 1:**
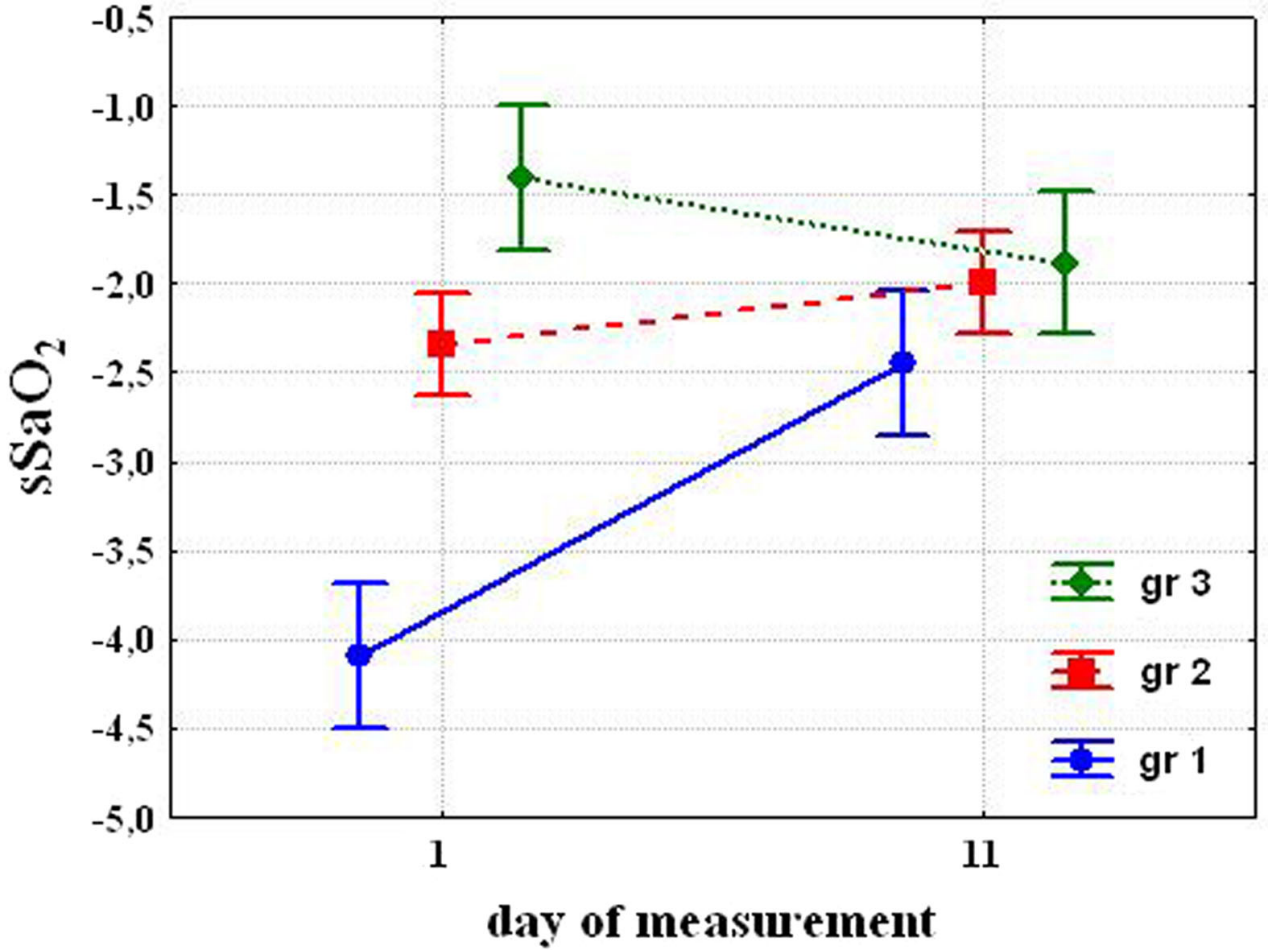
Changing susceptibility to hypoxia (sSaO_2_) after cold exposure.

Analysis of variances (MANOVA) shows the key role of susceptibility to hypoxia and cold exposures and their interrelation. Posterior analysis (Fisher LSD) showed that before cold exposures, susceptibility to hypoxia was significantly different in the selected groups. After cold acclimation susceptibility to hypoxia significantly decreased in the Group 1 (−2.45±0.24. P=0.008), tended to decrease in the Group 2 (−1.99±0.14; P=0.068) and tended to increase in the Group 3 (−1.88±0.21, P=0.09) (Fig. 1). While the susceptibility to hypoxia decreased almost double in Group 1, had a tendency to decrease in Group 2 and to increase in Group 3, the difference between the groups after 10 days was not significant.

**Fig. 2:**
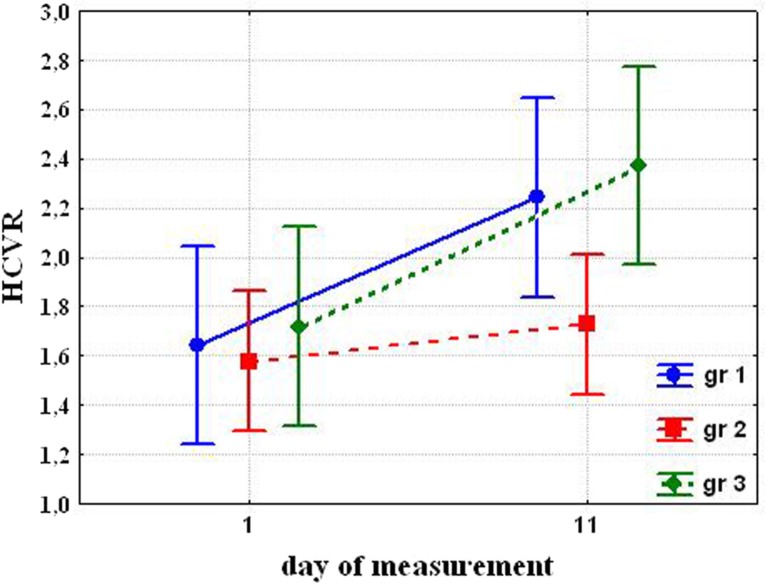
Changing hypercapnic ventilatory response (HCVR) after cold exposure.

Before cold exposures, HCVR and HVR were not significantly different in the groups. After cold acclimation, HCVR significantly increased in the Group 1 (1.64±0.065 before cold exposures vs. 2.24±0.153 after cold acclimation (P=0.039)) and Group 3 (1.72±0.121 vs. 2.37±0.26 (P=0.013)). While differences were not significant before exposure, Group 1 and 3 became significantly different from Group 2 (1.58±0.157 vs. 1.73±0.163 (NS) (Fig. 2).

**Fig. 3:**
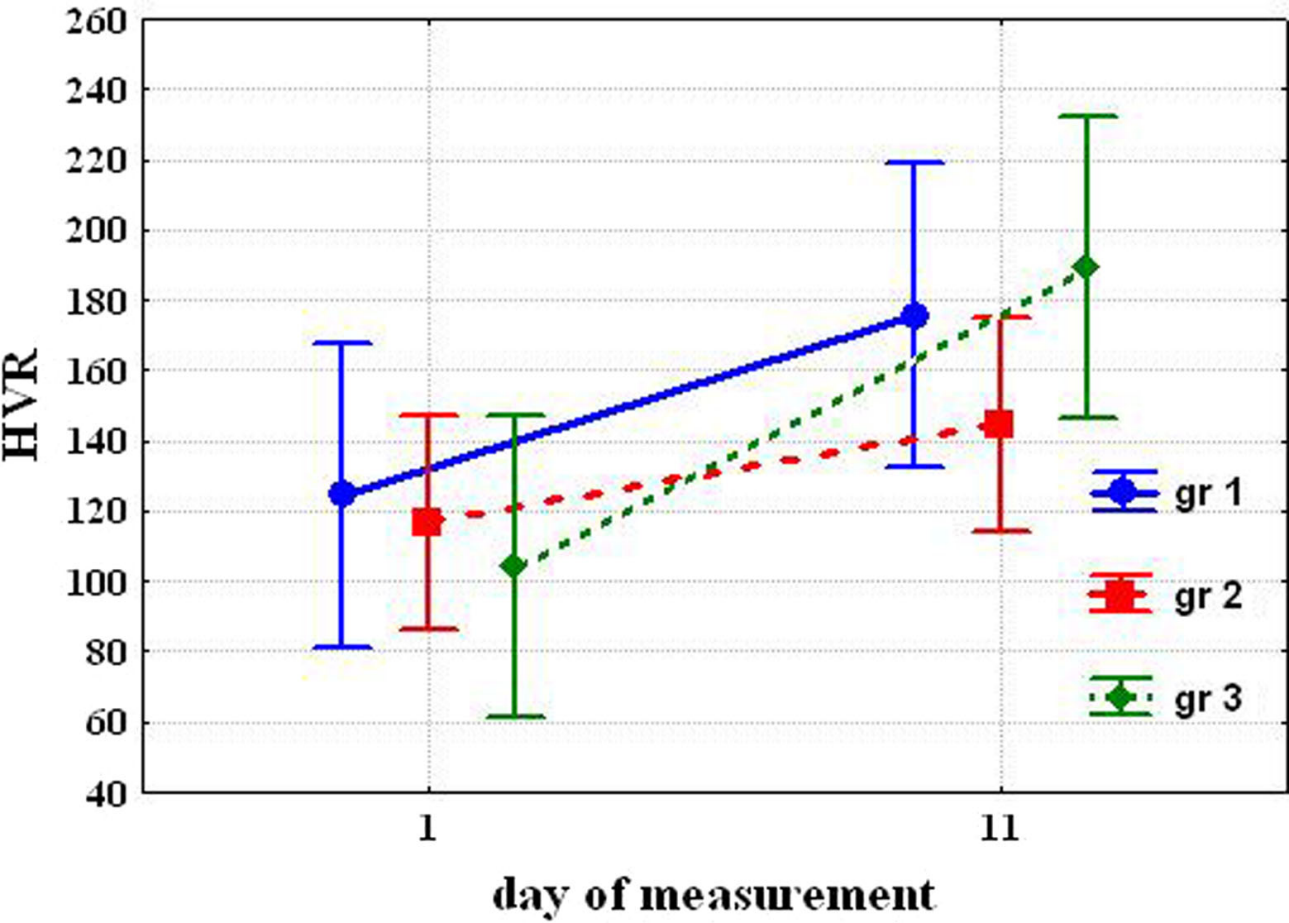
Changing hypoxic ventilatory response (HVR) after cold exposure.

After cold acclimation, HVR significantly increased only in Group 3 [104.0±16.23 vs. 189.5±27.08 (P=0.037)] and tended to increase in Group 1 [124.5±9.46 vs. 175.6±15.01 (NS)] and in Group 2 [116.9±13.12 vs. 144.9±19.94 (NS)] (Fig. 3).

The most significant changes in chemosensitivity to O_2_ and CO_2_ occurred in Group 3. In Group 1, HCVR significantly increased, and HVR tended to increase. In Group 2, HCVR did not change, and HVR tended to increase.

It should be mentioned that chemosensitivity to CO_2_ and O_2_ before cold exposures did not differ in the selected group. Savourey et al. (20) did not find any correlation between acute mountain sickness (AMS) and HVR. This indicates that ventilatory responses (HVR and HCVR) as parameters of breathing control may serve as additional information in the evaluation of susceptibility to hypoxia. Assessment of susceptibility to hypoxia may be helpful because physical activity and unusual environmental conditions may increase the risk of getting sick (4).

## Conclusions

After 10 days of cold exposure, all groups showed signs of hypothermic isoinsulative acclimation: decreased core temperature during cold test after cold acclimation and the same skin temperature at the end of cold tests in the first and tenth day.

Susceptibility to hypoxia decreased, which was accompanied by an increase in ventilatory response to CO_2_ and O_2_ that are typical responses to hypoxic exposure.

The individual susceptibility to hypoxia reflects interrelation of 3 self-regulated compartments, which are respiratory, cardiovascular systems and blood. The level of haemoglobin saturation (SaO_2_) is a result of this set of interactions. It can be a key parameter for estimating the hypoxic susceptibility. The more saturation slope in the hypoxic test, the more susceptibility to hypoxia and less tolerance to hypoxia.

Ventilatory responses (HVR and HCVR) as parameters of breathing control only may serve as additional information in the evaluation of susceptibility to hypoxia.

Individual group analysis revealed a variety in human respiratory control strategies. Group 1 (high susceptibility to hypoxia) increased the tolerance to hypoxia.

Changes in respiratory control occurred primarily due to changes in chemosensitivity to CO_2_. We conclude that hypoxia training would be highly effective for subjects from this group. The subjects in Group 3 (low susceptibility) showed most significant changes in the respiratory control, which indicates their strategy to maintain their state variables at the same level in spite of cold and hypoxia. It is possible that hypoxia training is not useful for them. Group 2 showed the most appropriate energy conservation reaction mode to cold exposures, which resulted in minimum changes in the respiratory control: HCVR and HVR did not change.

We plan to investigate the interrelation between the sensitivity to hypoxia and resistance to cold in further studies.
